# Supplementary material for: The People versus Behavioral Science: Alignment between lay and scientific understanding of compliance
Source: PLoS One. 2026 Jan 2;21(1):e0338675. doi: 10.1371/journal.pone.0338675 (PMC12758818; doi:10.1371/journal.pone.0338675)
Supplement: S1 File — (DOCX) [file pone.0338675.s001.docx]

**S1 File**

***Measures of compliance and behavioral mechanisms***

**Compliance** (based on Reinders Folmer et al., 2021)

I keep a safe distance (1.5 meters or more):

1. When I am indoors with others from outside of my direct household
2. When I am outdoors with others from outside of my direct household
3. From my neighbors
4. From colleagues at work
5. From friends and family from outside of my direct household
6. From others when grocery shopping
7. From others in the street
8. From others when taking a walk or exercising
9. From others in public transport

(1 = “never,” 7 = “always”)

**Rational choice mechanisms**

***Costs of compliance*** *(Van Rooij et al., 2020)*

Due to the measures to contain the coronavirus, I will probably:

1. Lose income
2. Lose my job
3. Not be able to work
4. Not be able to work as effectively as normal
5. Experience a negative impact on my social life

(1 = “extremely unlikely,” 7 = “extremely likely”)

***Benefits of compliance*** *(Van Rooij et al., 2020)*

I believe the coronavirus is a major threat to:

1. My own health
2. The health of friends and family
3. The general health

(1 = “strongly disagree,” 7 = “strongly agree”)

***Deterrence: punishment certainty*** *(Van Rooij et al., 2020)*

1. How likely is it that enforcement authorities will find out if you do not comply with this measure?
2. How likely is it that enforcement authorities will punish you if you do not comply with this measure?

(1 = “extremely improbable,” 7 = “extremely probable”)

***Deterrence: punishment severity*** *(Van Rooij et al., 2020)*

If enforcement authorities would punish you for not keeping a safe distance from people from outside of your own household:

1. How negative would the impact of this be for you?

(1 = “not at all negative;” 7 = “very strongly negative”)

**Social mechanisms**

***Descriptive social norms*** *(based on Reinders Folmer et al., 2021)*

Most people around me keep a safe distance from others (1.5 meters or more):

1. When they are indoors with others from outside of my direct household
2. When they are outdoors with others from outside of my direct household
3. From their neighbors
4. From colleagues at work
5. From friends and family from outside of their direct household
6. From others when grocery shopping
7. From others in the street
8. From others when taking a walk or exercising
9. From others in public transport

(1 = “strongly disagree,” 7 = “strongly agree;”)

**Legitimacy mechanisms**

***Moral alignment*** *(Van Rooij et al., 2020)*

1. I morally believe that people should keep a safe distance from others (1.5 meters or more) in order to contain the coronavirus

(1 = “strongly disagree,” 7 = “strongly agree”)

***Perceived effectiveness and proportionality***

The 1.5 meter measure to prevent the spread of the coronavirus is:

1. Effective
2. Proportionate to the severity of the pandemic
3. Proportionate to the costs that it inflicts

(1 = “strongly disagree,” 7 = “strongly agree”)

***Duty to obey the law*** *(Fine et al., 2016)*

It is acceptable to break a legal rule if:

1. The legal rule is clearly against your moral principles
2. This legal rule makes unreasonable demands of you
3. Obeying this legal rule is very expensive for you
4. This legal rule is not enforced
5. Most of your direct colleagues and/or friends also break this legal rule
6. You are in one way or another unable to do what this legal rule asks of you
7. Most of your direct colleagues and/or friends think breaking the legal rule is justified
8. You do not know this legal rule
9. You do not understand this legal rule
10. This legal rule has not been published
11. You feel that this legal rule was made without representing your interests
12. You think this legal rule is enforced unfairly

(1 = “strongly disagree,” 7 = “strongly agree”)

**Capacity mechanisms**

***Knowledge of the rules*** *(Reinders Folmer et al., 2021)*

1. Is there currently in your area an obligation to keep a safe distance (1.5 meter or more) from others from outside of your own household?

(1 = yes, 2 = no, 3 = don’t know)

***Understanding of the rules*** *(based on Van Rooij et al., 2020)*

The following questions are about the measure to keep a safe distance (1.5 meter or more) from others from outside of your own household. To what extent is it clear to you:

1. What this measure entails?
2. What this measure demands of you?
3. What you should do according to this measure?

(1 = “extremely unclear;” 7 = “extremely clear”)

***Practical capacity to comply*** *(based on Reinders Folmer et al., 2021)*

At this moment, I am capable of keeping a safe distance (1.5 meters or more) from others:

1. When I am indoors with others from outside of my direct household
2. When I am outdoors with others from outside of my direct household
3. From my neighbors
4. From colleagues at work
5. From friends and family from outside of my direct household
6. From others when grocery shopping
7. From others in the street
8. From others when taking a walk or exercising
9. From others in public transport

(1 = “completely disagree,” 7 = “completely agree”)

***Self-control*** *(Weinberger & Schwartz, 1990)*

1. I should try harder to control myself when I'm having fun (R)
2. I do things without giving them enough thought (R)
3. When I'm doing something fun (like partying or acting silly), I tend to get carried away and go too far (R)
4. I say the first thing that comes to my mind without thinking enough about it (R)
5. I stop and think things through before I act

(1 = “false,” 5 = “true”). (R) indicates reverse-coded item.

**Strain** *(Van Rooij et al., 2020)*

Due to coronavirus I feel:

1. Angry
2. Scared
3. Powerless
4. Depressed
5. Stressed
6. Lonely

(1 = “completely disagree,” 7 = “completely agree”)

**Opportunity mechanisms**

***Opportunity to violate*** *(based on Reinders Folmer et al., 2021)*

How often do you notice that it would still be possible to come within an unsafe distance (less than 1.5 meters) from others:

1. When I am indoors with others from outside of my direct household
2. When I am outdoors with others from outside of my direct household
3. From my neighbors
4. From colleagues at work
5. From friends and family from outside of my direct household
6. From others when grocery shopping
7. From others in the street
8. From others when taking a walk or exercising
9. From others in public transport

(1 = “never,” 7 = “always”)
