# Supplementary material for: The People versus Behavioral Science: Alignment between lay and scientific understanding of compliance
Source: PLoS One. 2026 Jan 2;21(1):e0338675. doi: 10.1371/journal.pone.0338675 (PMC12758818; doi:10.1371/journal.pone.0338675)
Supplement: S2 File — (DOCX) [file pone.0338675.s002.docx]

**Dataset and syntax files**

All relevant datasets and syntax files can be accessed at the Figshare repository of the University of Amsterdam through the following [link](https://figshare.com/s/e1b30a4c3ff30de3d3a1). Stored here are the following files:

|  | **File name** | **Description** | **Program** |
| --- | --- | --- | --- |
| 1 | Lay vs scientific understanding of compliance_DATA.dta | Full dataset (used for all individual-level analyses and to compute the aggregated (sample-level) values | STATA |
| 2 | Lay vs scientific understanding of compliance_SYNTAX imputations.do | Syntax (for full dataset; contains multiple imputation procedure, individual-level analyses, computation of aggregated (sample-level) values, and imputation checks) | STATA |
| 3 | Lay vs scientific understanding of compliance_AGGR DATA Comparison.sav | Aggregated dataset (used for comparison of lay understanding with empirical understanding) | SPSS |
| 4 | Lay vs scientific understanding of compliance_SYNTAX Comparison.sps | Syntax (for aggregated dataset; contains comparison of lay understanding with empirical understanding) | SPSS |
