# Supplementary material for: The People versus Behavioral Science: Alignment between lay and scientific understanding of compliance
Source: PLoS One. 2026 Jan 2;21(1):e0338675. doi: 10.1371/journal.pone.0338675 (PMC12758818; doi:10.1371/journal.pone.0338675)
Supplement: S3 File — (DOCX) [file pone.0338675.s003.docx]

**S3 File**

***Multiple imputation procedure, results, and checks***

**Imputation of missing values**

All analyses were conducted in Stata 16.0 SE [1]. Descriptive analyses indicated that nearly all cases featured missing data. The variables with the greatest proportion of missing data were compliance at work (42.1% missing) and in public transport (60.4% missing);^[[1]](#footnote-1)^ for all other variables, less than 7.4% of values were missing.^[[2]](#footnote-2)^ Because our previous research demonstrated strong internal consistency between the compliance items [2], we expected that these missing responses could be estimated from the other compliance items, as well as from additional relevant variables in the dataset, such as, employment (i.e., missing at random, MAR). As such, we imputed missing values by means of multiple imputation [see 3, 4]. We relied on multiple imputation by chained equations [MICE, see 5, 6]. Measures of lay understanding, policy preferences, and compliance and its theoretical mechanisms were included in the imputation model. Additionally, demographic and control variables were included as auxiliary variables [7], as were the supplementary (explorative) measures on other mitigation measures (see footnote 5). To accommodate the non-normal distribution of most variables, we used Predictive Mean Matching [PMM, see 8] for continuous variables; binary variables were estimated using logistic regression. In line with recent recommendations, imputation was conducted on the item (rather than scale) level [9]; scale means were computed afterwards based on the imputed data. Reliability statistics were obtained by computing alphas within each imputed dataset, and computing their pooled mean across imputations. Correlations of multiply imputed data were computed by means of the micorr command [10].

A trial run indicated a fraction of missing information (FMI) of .31 (95% CI = [0.20; 0.45]). Based on this, the HOW_MANY_IMPUTATIONS module [11] indicated that 42 imputations would be needed. As such, the final imputation model was estimated by creating 42 imputed datasets (10 burn-in iterations, 10 between-imputation iterations; 420 iterations in total).

Checks of the imputation model following the recommendations of Nguyen, Carlin and Lee (12) are presented below. In brief, the imputation checks revealed that for the variables with the highest proportion of missing responses (i.e., compliance at work and in public transport), the imputed data yielded a similar distribution as the observed data. However, especially for compliance at work, the checks revealed that compared with the observed data, the imputation model tended to impute values at the higher end of the range, and less frequently the (relatively less common) values at the lower end of the range. Given that these items were part of a scale for which the remaining items were largely complete (i.e., less than 7% missing data per item), we regard these discrepancies as relatively minor, and of limited impact on the overall compliance scale score. For comparison, the Appendix also provides descriptive statistics (Tables A1, A2, and A3) and regression results (Table A4) for two alternative treatments of the sample: (1) a sample of complete cases only, i.e., with complete responses for all measures including compliance at work and in public transport (N = 751); and (2) a sample of complete cases when omitting the items with the greatest proportion of missing data (i.e., compliance at work and in public transport) from the compliance scale measure (N = 2543). As the tables reveal, the results for these alternative samples were highly similar to the imputed data (albeit with lower statistical power). This strengthens our confidence in the findings based on the imputed data, which we report here.

**Table A1**

*Empirical understanding of compliance: Means and standard deviations of dependent and independent variables for empirical analysis of mechanisms shaping compliance (complete cases, missing omitted, and imputed data)*

| Mechanism | Complete cases (N = 751) | Missing items omitted (N = 2543) | Imputed data (N = 3326) |
| --- | --- | --- | --- |
| Compliance | 5.26 (1.35) | 5.57 (1.20) | 5.58 (1.16) |
| Costs of compliance | 3.36 (1.37) | 3.16 (1.28) | 3.13 (1.28) |
| Benefits of compliance (perceived threat) | 5.01 (1.51) | 5.19 (1.42) | 5.24 (1.43) |
| Punishment likelihood | 3.09 (1.53) | 2.83 (1.49) | 2.83 (1.50) |
| Punishment severity | 4.12 (1.71) | 4.05 (1.77) | 4.05 (1.81) |
| Social norms | 4.45 (1.28) | 4.56 (1.21) | 4.53 (1.23) |
| Moral alignment | 5.69 (1.49) | 5.91 (1.34) | 5.90 (1.36) |
| Perceived effectiveness and proportionality | 5.30 (1.51) | 5.50 (1.37) | 5.50 (1.38) |
| Obligation to obey the law | 4.58 (1.33) | 4.71 (1.26) | 4.66 (1.21) |
| Knowledge | 81.09% | 81.79% | 81.07% |
| Understanding | 6.23 (0.99) | 6.36 (0.84) | 6.36 (0.84) |
| Capacity to comply | 5.19 (1.10) | 5.36 (0.96) | 5.36 (0.97) |
| Impulsivity | 2.01 (0.81) | 1.91 (0.76) | 1.89 (0.76) |
| Negative emotions | 3.13 (1.52) | 2.90 (1.47) | 2.78 (1.40) |
| Opportunity to violate | 3.76 (1.42) | 3.55 (1.41) | 3.51 (1.43) |
| Age | 48.18 (14.99) | 53.48 (14.92) | 54.26 (14.85) |
| Trust in science | 3.95 (1.03) | 4.01 (0.96) | 4.01 (0.97) |
| Employed | 74.17% | 56.70% | 53.07% |
| Health risk self | 25.30% | 31.85% | 34.61% |

*Note*. Standard deviations between parentheses.

**Table A2**

*Lay understanding of compliance: Perceived associations of compliance mechanisms with compliance according to lay participants (complete cases, missing omitted, and imputed data)*^[[3]](#footnote-3)^

| Mechanism | Complete cases | Missing items omitted | Imputed data |
| --- | --- | --- | --- |
| 1. Costs of compliance | 4.92 (1.42) | 4.81 (1.41) | 4.80 (1.41) |
| 2. Benefits of compliance (perceived threat) | 5.59 (1.34) | 5.66 (1.24) | 5.63^a^ (1.26) |
| 3. Punishment | 4.57 (1.55) | 4.45 (1.53) | 4.41^b^ (1.56) |
| 4. Social norms | 5.30 (1.25) | 5.41 (1.19) | 5.38^c^ (1.23) |
| 5. Moral alignment | 5.38 (1.26) | 5.45 (1.21) | 5.42^d^ (1.24) |
| 6. Perceived effectiveness and proportionality | 5.35 (1.35) | 5.39 (1.29) | 5.36^e^ (1.32) |
| 7. Obligation to obey the law | 5.23 (1.30) | 5.23 (1.26) | 5.20^f^ (1.29) |
| 8. Knowledge | 5.39 (1.45) | 5.43 (1.43) | 5.41^g^ (1.45) |
| 9. Understanding | 5.35 (1.35) | 5.37 (1.31) | 5.36^a^ (1.32) |
| 10. Capacity to comply | 5.44 (1.22) | 5.48 (1.17) | 5.46^h^ (1.20) |
| 11. Impulsivity | 5.13 (1.37) | 5.19 (1.31) | 5.16^c^ (1.34) |
| 12. Negative emotions | 5.08 (1.39) | 5.02 (1.35) | 5.01^i^ (1.36) |
| 13. Opportunity to violate | 5.04 (1.32) | 5.02 (1.27) | 5.00^j^ (1.29) |

*Note*. Standard deviations between parentheses. Means with differing superscripts indicate significant differences at *p* < .004 (Bonferroni correction).

**Table A3**

*Lay understanding of compliance: Preference for policies leveraging main compliance mechanisms to promote compliance (complete cases, missing items omitted, and imputed data)*^[[4]](#footnote-4)^

| Mechanism | Complete cases | Missing items omitted | Imputed data |
| --- | --- | --- | --- |
| 1. Costs of compliance | 5.19 (1.55) | 5.24 (1.45) | 5.22^a^ (1.45) |
| 2. Benefits of compliance (perceived threat) | 5.46 (1.61) | 5.63 (1.48) | 5.63^e^ (1.50) |
| 3. Punishment | 3.85 (1.97) | 3.78 (1.94) | 3.78^f^ (1.94) |
| 4. Social norms | 5.26 (1.47) | 5.35 (1.40) | 5.33^i^ (1.42) |
| 5. Moral alignment | 5.26 (1.54) | 5.38 (1.48) | 5.37^b^ (1.50) |
| 6. Perceived effectiveness and proportionality | 5.60 (1.44) | 5.72 (1.34) | 5.73^c^ (1.36) |
| 7. Obligation to obey the law | 4.90 (1.69) | 5.09 (1.63) | 5.08^d^ (1.65) |
| 8. Knowledge | 4.95 (1.71) | 5.10 (1.63) | 5.12 (1.63) |
| 9. Understanding | 5.02 (1.68) | 5.21 (1.59) | 5.21^a^ (1.60) |
| 10. Capacity to comply | 5.44 (1.48) | 5.53 (1.34) | 5.53^g^ (1.36) |
| 11. Impulsivity | 4.62 (1.72) | 4.73 (1.65) | 4.73^h^ (1.66) |
| 12. Negative emotions | 5.24 (1.43) | 5.27 (1.39) | 5.27^j^ (1.39) |
| 13. Opportunity to violate | 4.60 (1.77) | 4.69 (1.71) | 4.73^h^ (1.71) |

*Note*. Standard deviations between parentheses. Means with differing superscripts indicate significant differences at *p* < .004 (Bonferroni correction).

**Table A4**

*Empirical understanding of compliance: Observed associations (unstandardized and standardized regression coefficients) of compliance mechanisms with compliance according to empirical analysis (complete cases, missing items omitted, and imputed data)*

| Mechanism | Model 1:  Complete cases (unstandardized) | Model 2:  Missing items omitted (unstandardized) | Model 3: imputed data (unstandardized) | Model 3: imputed data (standardized) |
| --- | --- | --- | --- | --- |
| Costs of compliance | .02 (.02) | .03 (.01) | .02 (.01) | .02 |
| Benefits of compliance (perceived threat) | .03 (.03) | .06*** (.02) | .06*** (.01) | .08 |
| Punishment likelihood | -.03 (.02) | -.01 (.01) | -.00 (.01) | -.00 |
| Punishment severity | .02 (.02) | .02* (.01) | .01 (.01) | .01 |
| Social norms | .12*** (.03) | .09*** (.02) | .07*** (.01) | .07 |
| Moral alignment | .18*** (.04) | .21*** (.03) | .20*** (.02) | .23 |
| Perceived effectiveness and proportionality | .11*** (.03) | .07*** (.02) | .08*** (.02) | .09 |
| Obligation to obey the law | .14*** (.03) | .08*** (.02) | .05*** (.01) | .05 |
| Knowledge | .19* (.09) | .12*** (.01) | .09* (.04) | .03 |
| Understanding | -.04 (.05) | .03 (.03) | .05* (.02) | .03 |
| Capacity to comply | .46*** (.05) | .44*** (.03) | .46*** (.02) | .38 |
| Impulsivity | -.08 (.04) | -.14*** (.02) | -.13*** (.02) | -.08 |
| Negative emotions | -.01 (.02) | .00 (.01) | .02* (.01) | .03 |
| Opportunity to violate | -.07*** (.02) | -.06*** (.01) | -.06*** (.01) | -.08 |
|  |  |  |  |  |
| **Controls** |  |  |  |  |
| Age | .00 (.00) | .00* (.00) | .00*** (.00) | .04 |
| Trust in science | -.01 (.04) | -.03 (.02) | -.04* (.02) | -.03 |
| Employed | .02 (.07) | -.00 (.04) | -.05 (.03) | -.02 |
| Health risk self | .12 (.07) | .11*** (.03) | .10*** (.03) | .04 |
|  |  |  |  |  |
| Constant | .23 (.37) | .45* (.21) | .59*** (.17) |  |
|  |  |  |  |  |
| **Rsq** | **.65** | **.58** | **.60** | **.60** |

**Imputation checks**

**Table A5**

Main missing data patterns (n = 3326)

| Percent | Compliance item 9 (public transport) | Compliance item 4 (colleagues at work) | Income | Compliance item 8 (walk or exercise) |
| --- | --- | --- | --- | --- |
| 15% | + | + | + | + |
| 17% | - | + | + | + |
| 15% | - | - | + | + |
| 7% | + | - | + | + |
| 7% | + | + | - | + |
| 7% | - | + | - | + |
| 4% | - | - | - | + |
| 2% | + | - | - | + |
| 1% | - | - | + | - |
| <1% | - | + | + | - |
| <1% | - | + | - | - |

Nb. + indicates value is present and − indicates value is missing. All other missing data patterns <1%.

**Table A6**

Baseline characteristics of participants with complete and incomplete data for the variables in the empirical understanding of compliance analysis

| Variable | Complete cases (n = 765) | Incomplete cases (n = 2549) |
| --- | --- | --- |
| Age, mean (SD) | 48.03 (15.00) | 56.11 (14.92) |
| Gender, fraction (%)  *Female*  *Male* | 403/765 (52.7)  362/765 (47.3) | 1425/2549 (55.9)  1124/2549 (44.1) |
| Education, fraction (%)  *None*  *Primary school*  *Lower secondary/preparatory secondary vocational*  *Higher secondary/secondary vocational*  *Higher vocational/university BA*  *Higher vocational/university MA* | 1/765 (0.1)  18/765 (2.4)  71/765 (9.3)  303/765 (39.6)  207/765 (27.1)  165/765 (21.6) | 2/2546 (0.1)  52/2546 (2.0)  387/2546 (15.2)  1091/2546 (42.9)  647/2546 (25.4)  367/2546 (14.4) |
| Employment, fraction (%) | 557/747 (74.6) | 1166/2498 (46.7) |
| Income, fraction (%)  *Minimum*  *Below average*  *Nearly average*  *Average*  *Between 1 and 2x average*  *2x average*  *More than 2x average* | 27/532 (5.1)  92/532 (17.3)  70/532 (13.2)  97/532 (18.2)  154/532 (28.9)  48/532 (9.0)  44/532 (8.3) | 105/1908 (5.5)  399/1908 (20.9)  283/1908 (14.8)  357/1908 (18.7)  490/1908 (25.7)  134/1908 (7.0)  140/1908 (7.3) |
| Conservatism, mean (SD) | 2.27 (0.70) | 2.42 (0.70) |
| Trust in science, mean (SD) | 3.94 (1.03) | 4.03 (0.95) |
| Care for COVID patients, fraction (%) | 109/766 (14.2) | 178/2551(7.0) |
| Health risk self, fraction (%) | 190/744 (25.5) | 930/2491 (37.3) |
| Health risk others, fraction (%) | 496/746 (66.5) | 1753/2492 (70.3) |
| Comply with facemask regulations, fraction (%) | 738/766 (96.3) | 2482/2547 (97.4) |
| Comply with self-isolation regulations, fraction (%) | 709/766 (92.6) | 2411/2549 (94.6) |
| Comply with testing regulations, fraction (%) | 664/766 (86.7) | 2244/2549 (88.0) |

Nb. The denominators in the fractions are the numbers of participants for whom the measure was available.

**Figure A1**

a

b

c

d

**Fig. A1** Graphs comparing the distributions of the observed (n = 1925) and imputed (n = 1401) scores for Compliance item 4 (safe distance from colleagues at work). (a) Kernel density plot of the observed (solid line) and imputed (dashed line) compliance scores, (b) histogram of the observed (transparent bars) and imputed (grey bars) compliance scores, (c) plot of the quantiles of the imputed compliance scores against quantiles of the observed scores (quantile–quantile plot), and (d) cumulative distribution plots of the observed (solid line) and the imputed (dashed line) compliance scores. The figure presents data from a single imputed dataset.

**Figure A2**

a

b

c

d

**Fig. A2** Graphs comparing the distributions of the observed (n = 1317) and imputed (n = 2009) scores for Compliance item 9 (safe distance while going for a walk or exercising). (a) Kernel density plot of the observed (solid line) and imputed (dashed line) compliance scores, (b) histogram of the observed (transparent bars) and imputed (grey bars) compliance scores, (c) plot of the quantiles of the imputed compliance scores against quantiles of the observed scores (quantile–quantile plot), and (d) cumulative distribution plots of the observed (solid line) and the imputed (dashed line) compliance scores. The figure presents data from a single imputed dataset.

**Figure A3**

**Fig. A3** Boxplots of the observed (labelled 0) and imputed (labelled 1–20) scores for Compliance item 4 (safe distance from colleagues at work). Data are shown for the first 20 imputed datasets.

**Figure A4**

**Fig. A4** Boxplots of the observed (labelled 0) and imputed (labelled 1–20) scores for Compliance item 9 (safe distance while going for a walk or exercising). Data are shown for the first 20 imputed datasets.

**Table A6**

Summary statistics of the observed and imputed data for the incomplete variables with greatest proportion of missing responses

| Variable | Observed | | | | | Imputed | | | | |
| --- | --- | --- | --- | --- | --- | --- | --- | --- | --- | --- |
|  | N | Mean | SD | Min | Max | N | Mean | SD | Min | Max |
| Compliance item 9 (public transport) | 1317 | 5.49 | 1.58 | 1 | 7 | 2009 | 5.66 | 1.40 | 1 | 7 |
| Compliance item 4 (colleagues at work) | 1925 | 5.15 | 1.65 | 1 | 7 | 1401 | 5.69 | 1.33 | 1 | 7 |
| Income | 2448 | 3.93 | 1.63 | 1 | 7 | 878 | 3.85 | 1.62 | 1 | 7 |
| Compliance item 8 (walk or exercise) | 3079 | 5.97 | 1.28 | 1 | 7 | 247 | 5.77 | 1.53 | 1.05 | 7 |
| Compliance item 1 (indoors non direct household) | 3175 | 4.98 | 1.86 | 1 | 7 | 151 | 5.60 | 1.55 | 1 | 7 |
| Political orientation | 3190 | 2.38 | 0.70 | 1 | 4 | 136 | 2.42 | 0.69 | 1 | 4 |
| OOL item 7 (most colleagues and/or friends think rule breaking is justified) | 3203 | 5.58 | 1.47 | 1 | 7 | 123 | 5.61 | 1.45 | 1.05 | 7 |

The summary statistics of the imputed data were calculated using pooled data over 42 imputations. SD standard deviation, Min minimum, Max maximum.

**Figure A5**

**Fig. A5** Scatterplot of the scores for Compliance item 4 (safe distance from colleagues at work) against the estimated probabilities of response with lowess curves. Data shown are observed values (black) and imputed values (red) for one imputed dataset only.

**Figure A6**

 **Fig. A6** Scatterplot of the scores for Compliance item 9 (safe distance while going for a walk or exercising) against the estimated probabilities of response with lowess curves. Data shown are observed values (black) and imputed values (red) for one imputed dataset only.

**Figure A7**

**Fig. A7** Plot of the residuals against the predicted values for the proposed imputation model fitted to the observed data for Compliance item 4 (safe distance from colleagues at work).

**Figure A8**

**Fig. A8** Plot of the residuals against the predicted values for the proposed imputation model fitted to the observed data for Compliance item 9 (safe distance while going for a walk or exercising).

**Figure A9**

**Fig. A9** Leave-one-out cross-validation plot for the scores for Compliance item 4 (safe distance from colleagues at work). The median imputed values across 20 imputations (black markers) have been plotted against the observed value. The error bars are the intervals between the 5th and 95th percentiles.

**Figure A10**

**Fig. A10** Leave-one-out cross-validation plot for the scores for Compliance item 9 (safe distance while going for a walk or exercising). The median imputed values across 20 imputations (black markers) have been plotted against the observed value. The error bars are the intervals between the 5th and 95th percentiles.

**References**

1. StataCorp. Stata Statistical Software: Release 16. College Station, TX: StataCorp LLC. 2019.

2. Reinders Folmer C, Brownlee M, Fine A, Kuiper ME, Olthuis E, Kooistra EB, et al. Social Distancing in America: Understanding Long-term Adherence to Covid-19 Mitigation Recommendations. Plos One. 2021;16(9):e0257945. doi: <https://doi.org/10.1371/journal.pone.0257945>.

3. Schafer JL, Graham JW. Missing data: our view of the state of the art. Psychological Methods. 2002;7(2):147-77. doi: 10.1037/1082-989X.7.2.147.

4. Graham JW. Missing data analysis: Making it work in the real world. Annual Review of Psychology. 2009;60:549-76. doi: 10.1146/annurev.psych.58.110405.085530.

5. Lee KJ, Carlin JB. Multiple imputation for missing data: fully conditional specification versus multivariate normal imputation. American Journal of Epidemiology. 2010;171(5):624-32.

6. White IR, Royston P, Wood AM. Multiple imputation using chained equations: issues and guidance for practice. Statistics in Medicine. 2011;30(4):377-99.

7. Collins LM, Schafer JL, Kam C-M. A comparison of inclusive and restrictive strategies in modern missing data procedures. Psychological Methods. 2001;6(4):330-51.

8. Vink G, Frank LE, Pannekoek J, Van Buuren S. Predictive mean matching imputation of semicontinuous variables. Statistica Neerlandica. 2014;68(1):61-90.

9. Gottschall AC, West SG, Enders CK. A comparison of item-level and scale-level multiple imputation for questionnaire batteries. Multivariate Behavioral Research. 2012;47(1):1-25.

10. Eddings W, Marchenko Y. A correlation matrix after multiple imputation. Accessed at: <https://www.stata.com/statalist/archive/2010-07/msg01382.html>. 2010.

11. von Hippel P. HOW_MANY_IMPUTATIONS: Stata module to determine required number of imputations. 2018.

12. Nguyen CD, Carlin JB, Lee KJ. Model checking in multiple imputation: an overview and case study. Emerging Themes in Epidemiology. 2017;14(1):1-12. doi: 10.1186/s12982-017-0062-6.

1. Missing responses on these variables mostly concern people who selected the “does not apply”-option. [↑](#footnote-ref-1)
2. For the control variable income, 26.4% of responses were missing. As this variable did not correlate significantly with compliance, we do not discuss it in detail in the main text. [↑](#footnote-ref-2)
3. Because the selection of cases for the non-imputed data (complete cases and missing items omitted) was based on the variables that were included in the regression model, it was possible for these participants to show some missing responses on subsequent measures, such as those of behavioral intuitions and instruments. As such, the means displayed here were based on the following sample sizes. Complete cases – items 2, 3, 13, 16, 19, 24: *N* = 751; items 1, 4, 6, 9, 11, 14, 15, 18, 21-23, 26: *N* = 750; items 5, 7, 8, 10, 12, 17, 20, 22, 25: *N* = 749. Missing items omitted (7-item compliance measure): items 3, 13, 19: *N* = 2543; items 2, 15, 16, 18, 21, 22, 24: *N* = 2542; items 4, 5, 6, 10, 11, 14, 20, 23, 26: *N* = 2541; items 1, 7-9, 12, 25: *N* = 2540; item 17: *N* = 2539. The imputed data are based on all 3326 cases. [↑](#footnote-ref-3)
4. Because the selection of cases for the non-imputed data (complete cases and missing items omitted) was based on the variables that were included in the regression model, it was possible for these participants to show some missing responses on subsequent measures, such as those of behavioral intuitions and instruments. As such, the means displayed here were based on the following sample sizes. Complete cases – items 2, 3, 13, 16, 19, 24: *N* = 751; items 1, 4, 6, 9, 11, 14, 15, 18, 21-23, 26: *N* = 750; items 5, 7, 8, 10, 12, 17, 20, 22, 25: *N* = 749. Missing items omitted (7-item compliance measure): items 3, 13, 19: *N* = 2543; items 2, 15, 16, 18, 21, 22, 24: *N* = 2542; items 4, 5, 6, 10, 11, 14, 20, 23, 26: *N* = 2541; items 1, 7-9, 12, 25: *N* = 2540; item 17: *N* = 2539. The imputed data are based on all 3326 cases. [↑](#footnote-ref-4)
