# Supplementary material for: The People versus Behavioral Science: Alignment between lay and scientific understanding of compliance
Source: PLoS One. 2026 Jan 2;21(1):e0338675. doi: 10.1371/journal.pone.0338675 (PMC12758818; doi:10.1371/journal.pone.0338675)
Supplement: S5 File — (DOCX) [file pone.0338675.s005.docx]

**S5 File**

***Standardized regression results***

**Regression analysis**

The results of the correlation analysis (Table 5) indicated many of the compliance mechanisms to show substantial intercorrelations. To assess their unique contributions to compliance, a regression model (with robust standard errors) was estimated (Table 8). In this model, all behavioral mechanisms were entered as the predictors. The model controlled for the all control variables that showed substantive significant correlations with compliance: age, trust in science, employment, and own health risk. The same eleven behavioral mechanisms emerged as significant predictors of compliance: (1) benefits of compliance (rational choice theories), (2) social norms (social theories), (3) moral alignment (legitimacy theories), (4) perceived effectiveness and proportionality (legitimacy theories), (5) duty to obey the law (legitimacy theories), (6) knowledge of the rules (capacity theories), (7) understanding of the rules (capacity theories), (8) practical capacity to comply (capacity theories), (9) impulsivity (self-control from capacity theories), (10) negative emotions (strain from capacity theories), and (11) opportunities for offending (opportunity theories). These results again globally concord with previous regression results on the associations of these mechanisms with compliance in research that used the same materials (see S4 File). To enable their relative contribution to this outcome to be directly compared, standardized regression coefficients were computed. Again, practical capacity to comply (from capacity theories) showed the strongest association with compliance (*β* = .38), while punishment likelihood (deterrence from rational choice theories) showed the weakest (*β* = -.00). However, although several mechanisms showed significant associations with compliance, these associations were minimal compared to that of capacity or (to a lesser extent) moral alignment. As such, when zooming in on their unique associations, compliance was principally associated with capacity to comply, and to a lesser extent, moral alignment with mitigation measures.

**Table 8. Empirical understanding of compliance: Observed associations (unstandardized and standardized regression coefficients) of compliance mechanisms with compliance according to empirical analysis.**

| **Mechanism** | **Unstandardized** | **Standardized** |
| --- | --- | --- |
| Costs of compliance | .02 (.01) | .02 |
| Benefits of compliance (perceived threat) | .06*** (.01) | .08 |
| Punishment likelihood | -.00 (.01) | -.00 |
| Punishment severity | .01 (.01) | .01 |
| Social norms | .07*** (.01) | .07 |
| Moral alignment | .20*** (.02) | .23 |
| Perceived effectiveness and proportionality | .08*** (.02) | .09 |
| Duty to obey the law | .05*** (.01) | .05 |
| Knowledge | .09* (.04) | .03 |
| Understanding | .05* (.02) | .03 |
| Capacity to comply | .46*** (.02) | .38 |
| Impulsivity | -.13*** (.02) | -.08 |
| Negative emotions | .02* (.01) | .03 |
| Opportunity to violate | -.06*** (.01) | -.08 |
|  |  |  |
| **Controls** |  |  |
| Age | .00*** (.00) | .04 |
| Trust in science | -.04* (.02) | -.03 |
| Employed | -.05 (.03) | -.02 |
| Health risk self | .10*** (.03) | .04 |
|  |  |  |
| Constant | .59*** (.17) |  |
|  |  |  |
| **Rsq** | **.60** | **.60** |

**Comparing lay understanding and empirical evidence**

To compare lay perceptions of how the different compliance mechanisms shape compliance with the empirically observed associations as shown by the regression results, we again followed the approach of Willoughby, Love [46]. That is, we again compared the (sample-level, aggregated) mean perceived influence scores for each of the 13 compliance mechanisms with the empirically observed associations between those mechanisms and compliance, as indicated by the standardized regression coefficients.


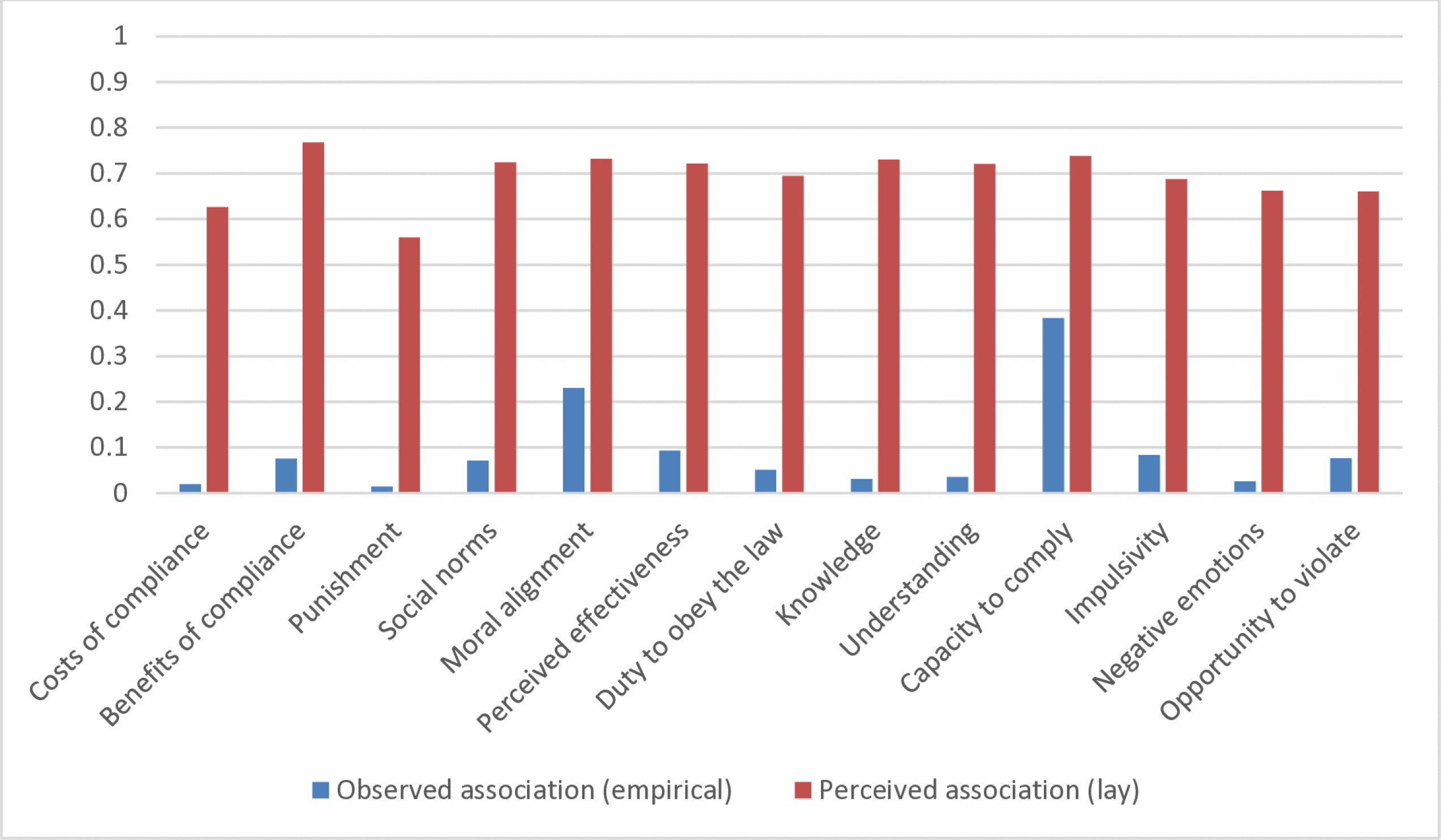


**Fig 5. Comparing lay and empirical understanding of compliance: Observed empirical associations (absolute standardized regression coefficient) of compliance mechanisms with compliance versus (aggregated) perceived associations with compliance according to lay participants.**


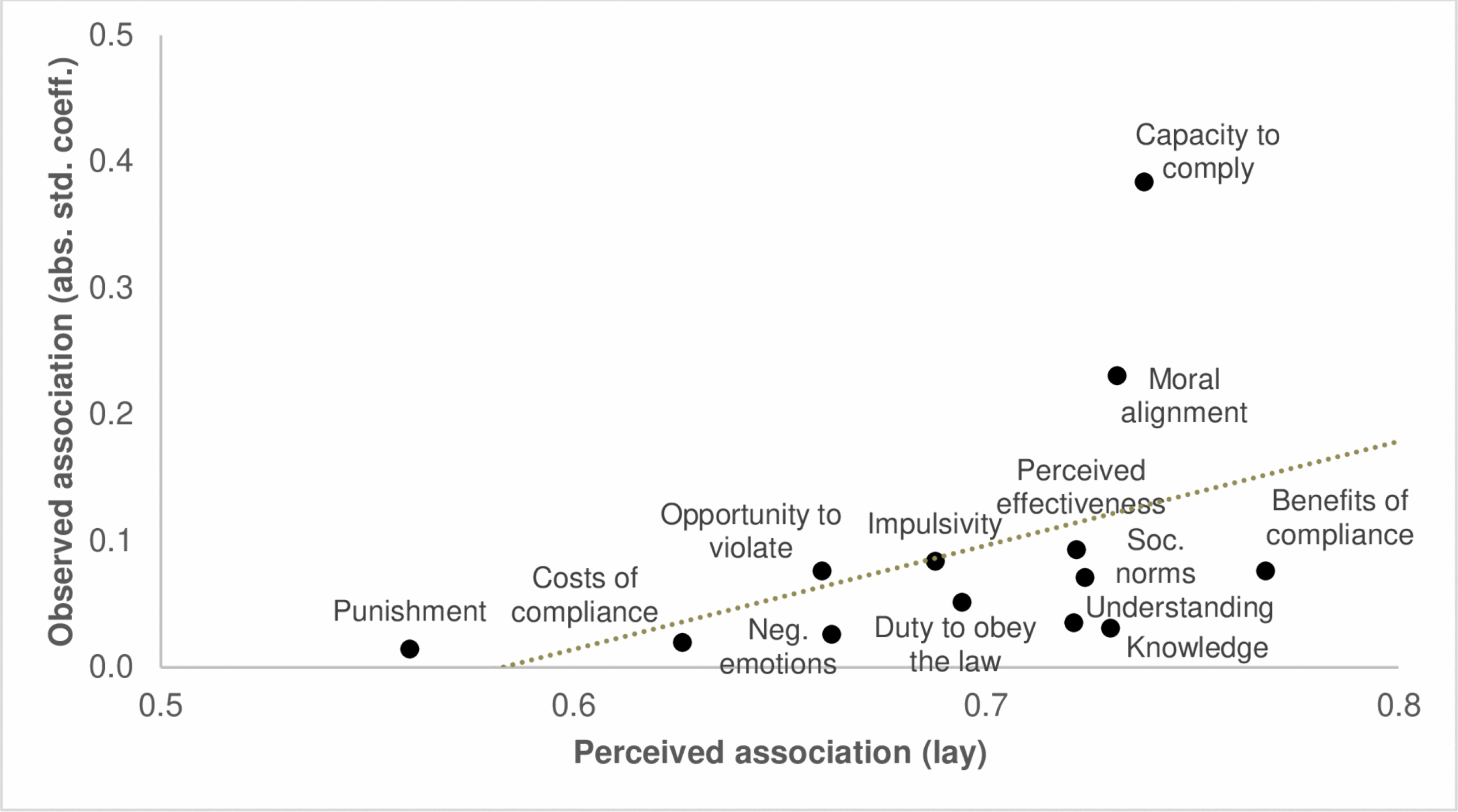


**Fig 6. Comparing lay and empirical understanding of compliance: Observed empirical associations (absolute standardized regression coefficient) of compliance mechanisms with compliance versus (aggregated) perceived associations with compliance according to lay participants (lay understanding).**

Figs. 5 and 6 demonstrate that this picture became more skewed when (mean) lay perceptions of the influence of the different mechanisms on compliance were compared to the (absolute) observed standardized regression coefficients, which control for the overlap between the predictors. Participants did on average perceive capacity to comply and moral alignment to be among the most influential mechanisms for compliance, which indeed ranked amongst the most influential mechanisms according to their observed associations. However, they did not on average recognize that these mechanisms had far stronger empirical associations with compliance compared with all other compliance mechanisms, as indicated by their standardized regression coefficients. Indeed, empirically, most other mechanisms showed only weak associations with compliance, and several mechanisms that were seen as relatively influential by lay participants ranked as moderate (e.g., benefits of compliance, social norms) or even weak in terms of their empirical associations (e.g., knowledge, understanding). This was also reflected by the Coefficient of Variation: (mean) lay perceptions of the influence of the different compliance mechanisms showed substantially less variability (CV_intuitions_ = 6.29) than the empirically observed associations, as indicated by the (absolute) standardized regression coefficients (CV_coefficients_ = 113.07). In line with this, computing the Spearman rank correlation between (mean) lay perceptions (average perceived influence scores for each of the 13 mechanisms) and the observed (absolute) standardized regression coefficients indicated a considerably reduced (but still large) correlation (*r* = .61). In sum, on average, participants perceived the influence of the different compliance mechanisms on compliance to be relatively strong and low in variability. This contrasted even more starkly with the empirically observed associations when looking at the standardized regression results.
